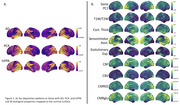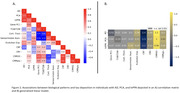# Subtype‐specific patterns of tau pathology in Alzheimer’s and related disorders

**DOI:** 10.1002/alz.086138

**Published:** 2025-01-03

**Authors:** Diana A Hobbs, Stephanie Doering, Austin A. McCullough, Peter R Millar, Shaney Flores, Sarah J. Keefe, Aristeidis Sotiras, Tammie L.S. Benzinger, Gregory S Day, Brian A. Gordon

**Affiliations:** ^1^ Washington University in St. Louis, St. Louis, MO USA; ^2^ Washington University in St. Louis School of Medicine, St. Louis, MO USA; ^3^ Washington University School of Medicine, St. Louis, MO USA; ^4^ Mayo Clinic, Jacksonville, FL USA

## Abstract

**Background:**

Alzheimer disease (AD) involves neurodegenerative disorders with progressive cognitive decline. Atypical presentations like Posterior Cortical Atrophy (PCA) and Logopenic Variant Primary Progressive Aphasia (lvPPA) exhibit distinct clinical profiles. PCA affects the posterior parietal and occipital lobes, causing visuospatial deficits, while lvPPA manifests as language impairment in the temporoparietal region. Understanding the biological underpinnings of these variants is crucial for deciphering AD’s heterogeneity.

**Method:**

Participants diagnosed with AD dementia (n = 54, female = 25, Age: 75.23±6.52), PCA (n = 9, female = 8, Age: 62.78±7.21), and lvPPA (n = 6, female = 2, Age: 66.33±6.38) from the Knight Alzheimer Disease Research Center (Knight ADRC) underwent ^18^F‐AV‐1451 tau‐PET imaging. Tau deposition, along with nine biological properties, was mapped to the cortical surface (Figure 1A): gene expression, myelin, cortical thickness, sensorimotor association axis, evolutionary expansion, cerebral blood flow (CBF), cerebral blood volume (CBV), and cerebral metabolic rate of glucose (CMRglu) (Figure 1B). Correlation matrices and generalized linear models (GLMs) assessed relationships between tau accumulation and biological properties.

**Result:**

A correlation matrix (Figure 2A) shows significant positive associations (p<0.05) between evolutionary expansion patterns and tau accumulation in AD (r = 0.53), PCA (r = 0.29), and lvPPA (r = 0.56); and gene expression in PCA (r = 0.51). Conversely, strongest negative associations were observed in AD (r = ‐0.38, r = ‐0.15) and lvPPA (r = ‐0.18, r = ‐0.28) for CBF and myelin mapping, respectively, and in PCA (r = ‐0.25, ‐0.23) for cortical thickness and sensorimotor association axis. GLMs demonstrated significant relationships between cerebrovascular health and evolutionary expansion with tau measures across all groups. AD (r = ‐0.52) and PCA (r = ‐0.19) exhibited negative associations with gene expression and myelin mapping, respectively. Additionally, lvPPA (r = 0.39) displayed positive association with the sensorimotor association cortex.

**Conclusion:**

Results elucidate distinct tau deposition patterns in AD, PCA, and lvPPA, providing insights into subtype‐specific pathophysiology. The correlation matrix and GLMs highlight the significance of evolutionary expansion and cerebrovascular health in tau accumulation. Associations underscore the complex interplay of gene expression, myelin mapping, and cortical thickness with tau pathology. Findings contribute to understanding heterogeneity within AD spectrum disorders, emphasizing the multifaceted nature of neurodegenerative processes. Further research promises refined diagnostic and therapeutic strategies tailored to distinct neurobiological profiles in AD, PCA, and lvPPA.